# Effect of appropriate extenders to maintain sperm functionality during short-term storage of sterlet (*Acipenser ruthenus*) sperm with fertilization assay under hatchery conditions

**DOI:** 10.1007/s10695-024-01413-7

**Published:** 2024-12-02

**Authors:** Nururshopa Eskander Shazada, Mohammad Abdul Momin Siddique, Songpei Zhang, Zhijun Ma, Marek Rodina, Otomar Linhart

**Affiliations:** 1 Faculty of Fisheries and Protection of Waters, South Bohemian Research Center of Aquaculture and Biodiversity of Hydrocenoses, Research Institute of Fish Culture and Hydrobiology, University of South Bohemia in České Budějovice, Zátiší 728/II, Vodňany, 38925 Czech Republic; 2 Department of Biotechnology and Genetic Engineering, Noakhali Science and Technology University, Noakhali, 3814 Bangladesh; 3 Department of Oceanography, Noakhali Science and Technology University, Noakhali, 3814 Bangladesh

**Keywords:** Sperm short-term storage, CASA, Sperm motility, Sperm velocity, Fertilization rate, Hatching rate

## Abstract

Short-term storage and management of sperm in vitro is an easy and economical process in which suitable extenders can be utilized to extend the storage period and prevent sperm function impairment. Therefore, the current study aimed to evaluate the effect of suitable extenders during the short-term storage of sterlet sperm and determine their fertilizing capacity and hatching success. Three extenders containing a composition of 16, 20, and 24 mM NaCl, 1 mM KCl, 0.1 mM CaCl_2_, 10 mM Tris, pH 8.0 with osmolarity of 46, 55, and 62 mOsm/kg, were used to dilute the sperm of four sexually mature sterlet males (*n* = 4). Using a CASA system, the motility and velocity of undiluted and diluted sperm with extenders (E1 – E3) were assessed over 6 days at 0–2 °C. The short-term stored diluted sperm was then used in the fertilization and hatching assay, and undiluted fresh and stored sperm was used as a control. A two-way factorial analysis of variance (ANOVA) model confirmed significant effects on sperm motility, curvilinear velocity (VCL), and straight-line velocity (VSL) (*P* < 0.001), as well as their interaction with the extender. The model was decomposed into a one-way ANOVA to examine the impacts of extenders and storage time. With increasing storage periods, the sperm motility and velocity gradually decreased for diluted sperm with three extenders (E1–E3) but sharply decreased for undiluted sperm (Control). The motility of undiluted sperm was found 3.77 ± 4.09% at 4 days, whereas sperm diluted with extenders showed 57.57 ± 12.33% (E1), 64.34 ± 11.86% (E2), and 61.40 ± 12.41% (E3) motility at 6 days. This study explored extenders optimized with higher osmolarity (39–62 mOsm/kg) and lower K^+^ (1 mmol/L) as the most suitable medium for storing sterlet sperm for 6 days. After 6 days post storage, sperm diluted with extenders E1–E3 achieved a fertilization rate of 31.29 ± 14.2%, 31.66 ± 8.84%, and 30.67 ± 10.02%, respectively, and hatching success of 29.58 ± 13.4%, 30.50 ± 7.89%, and 27.95 ± 9.62%, respectively with freshly ovulated eggs.

**Supplementary information:**

The online version contains supplementary material available at 10.1007/s10695-024-01413-7.

## Introduction

Sterlet (*Acipenser ruthenus*) is a member of the sturgeon family (Acipenseridae). It is a relatively small species among the group (maximum length 100 to 125 cm), but it is a scientifically and economically important species because of its fast growth and early age of maturity and ability to produce quality flesh and caviar and generate hybrids with other sturgeon species (Hochleithner and Gessner 2012). Due to overfishing and environmental deterioration, the stocks of wild populations of sturgeons have been steadily declining, making almost all these species threatened or endangered (Alavi et al. 2012; Tavakoli et al. 2021). Gamete conservation in fish farming has recently attracted much attention, particularly for economically significant and endangered species (Magnotti et al. 2018). Thus, the global demand for caviar and boneless flesh for human diets and conservation efforts to maintain the species has increased the development of sturgeon aquaculture (Bronzi et al. 2011).

Short-term storage and management of sperm are essential for captive breeding, which is a straightforward, affordable, and widely employed procedure with the following advantages: it keeps the sperm available when sexual maturity is asynchronous and induces in vitro maturation of sperm from sex-reversed male fishes. This helps transport sperm for long distances and reduces the need to keep broodstock available for an extended period (Contreras et al. 2020; Shazada et al. 2024a). Undiluted sturgeon sperm, including sterlet sperm, can maintain sperm functionality in an aerobic environment on ice (0 to 4 °C) for 2 to 6 days (Dettlaff et al. 1993). Maintaining sperm motility performance is crucial in determining fertility, which is improved when sperm are stored in an extender with seminal plasma properties (Park and Chapman 2005; Xin et al. 2020). The seminal plasma of low-quality sperm (< 20% motility) contains substantially lower amounts of Na^+^, K^+^, Cl^−^, total protein, and osmolality than the seminal plasma of high-quality sperm (> 80% motility), suggesting that incubation of sperm with good seminal plasma improves sperm motility and velocity for 96-h short-term storage and has a positive influence on fertilizing ability (Xin et al. 2020). Artificial seminal plasma or immobilized medium has typically been used as extenders to extend the storage period and prevent sperm function impairment. A previous study developed 20 extenders based on seminal plasma parameters with the manipulation of Na^+^ and K^+^. It optimized the three best extenders containing 16, 20, and 24 mM NaCl, 1 mM KCl, 0.1 mM CaCl2 10 mM Tris, and pH 8.0 with osmolality higher than that of seminal plasma to store sterlet sperm for 144 h (Shazada et al. 2024b).

The fertilization capacity and hatching rate of short-term storage of sterlet sperm in vitro are important for breeders. The fertilization capacity and reproductive success of males generally depend on sperm quantity (e.g. volume and concentration) and sperm quality (e.g. motility, seminal plasma composition, pH, and osmolarity) (Gage et al. 2004; Ciereszko et al. 2010). Sturgeon eggs have recently been incubated in Petri dishes with controlled temperature settings that did not allow water to flow through (Fatira et al. 2018). Shazada et al. (2024b) observed that sperm diluted with selected extenders retained sperm motility and velocity better than undiluted sperm. However, they did not determine the fertilization and hatching success of the short-term stored sperm diluted with optimized extenders. Therefore, the main aim of the present study was to determine the effect of selected extenders during the short-term storage of sterlet sperm and assess their fertilizing capacity and hatching success. The study’s specific objectives were (1) to investigate the motility and velocity of undiluted and diluted sperm within the storage period and (2) to determine fertilization, hatching, and malformation rate of the short-term stored and fresh sperm of sterlet.

## Materials and methods

### Ethical statement and experimental animals.

All experiments were conducted at the Faculty of Fisheries and Protection of Waters (FFPW), University of South Bohemia in České Budějovice (USB), Vodňany, Czech Republic, and they maintained the principles of the EU-harmonized Animal Welfare Act of the Czech Republic. Animal manipulations were performed following approval from the Czech Ministry of Agriculture for producing and distributing experimental animals according to Reference numbers 56665/2016-MZE-17214 and 64,155/2020-MZE-18134. Furthermore, FFPW, USB was permitted to utilize experimental animals (Reference number: 68763/2020-MZE-18134). Following Sect. 15d (3) of Act No. 246/1992 Coll. on the Protection of Animals Against Cruelty, the experimental advisor and the supervisor of this study are certified professionals who can design experiments and experimental projects.

### Broodstock husbandry and gamete collection

Five sexually mature sterlet males (3–7 years old and 1–3 kg body weight) and five females (4–12 years old and 2–3 kg body weight) were collected from the research facility center of the Faculty of Fisheries and Protection of Waters, University of South Bohemia at Vodnany, Czech Republic. In the preliminary stage before experimentation, sterlet broodstocks were kept in a 4 m^3^ hatchery tank with a water temperature of 15 °C, a water flow rate of 0.2 L s^−1^, and an oxygen content of 7.0 mg/L.

After acclimatization, males were induced by injecting a single intramuscular injection of carp pituitary (CP; Fish Farm Pohořelice Ltd, Czech Republic) dissolved in 0.9% (*w/v*) NaCl solution at a dose of 4 mg/kg body weight to induce spermiation. The sperm was collected using a plastic catheter (4-mm diameter) 48 h after hormonal treatment, stored in a 50 mL plastic container, and kept immediately on ice under aerobic conditions. Then, initial sperm motility was examined using a microscope, and semen with a minimal requirement of ˃80% motility was selected for short-term storage.

After acclimatization in the hatchery, ovulation of females was induced by injecting two doses of carp pituitary (CP; Fish Farm Pohořelice Ltd, Czech Republic) dissolved in 0.9% (*w/v*) NaCl solution; 0.5 mg/kg body weight was given as a priming dose and a second injection of 4.5 mg/kg body weight was administered 12 h after the priming dose. Following the second injection, 18–20 h later, the eggs were stripped into a plastic bowl by gentle abdominal massage (Gela et al. 2008; Siddique et al. 2016). The eggs were kept under aerobic conditions at 17 °C in an incubator with a cover which maintained the proportion of eggs and air at 30% and 70%. During egg stripping, females were handled carefully, and the standard laboratory protocol was maintained so as not to injure the fish from whom eggs were collected and to avoid contamination by body fluid. The darker the color was, the more suitable were the eggs. Eggs used in fertilization and hatching were selected from those not undergoing decomposition.

### Preparation of extenders and experimental design

**Fig. 1:**
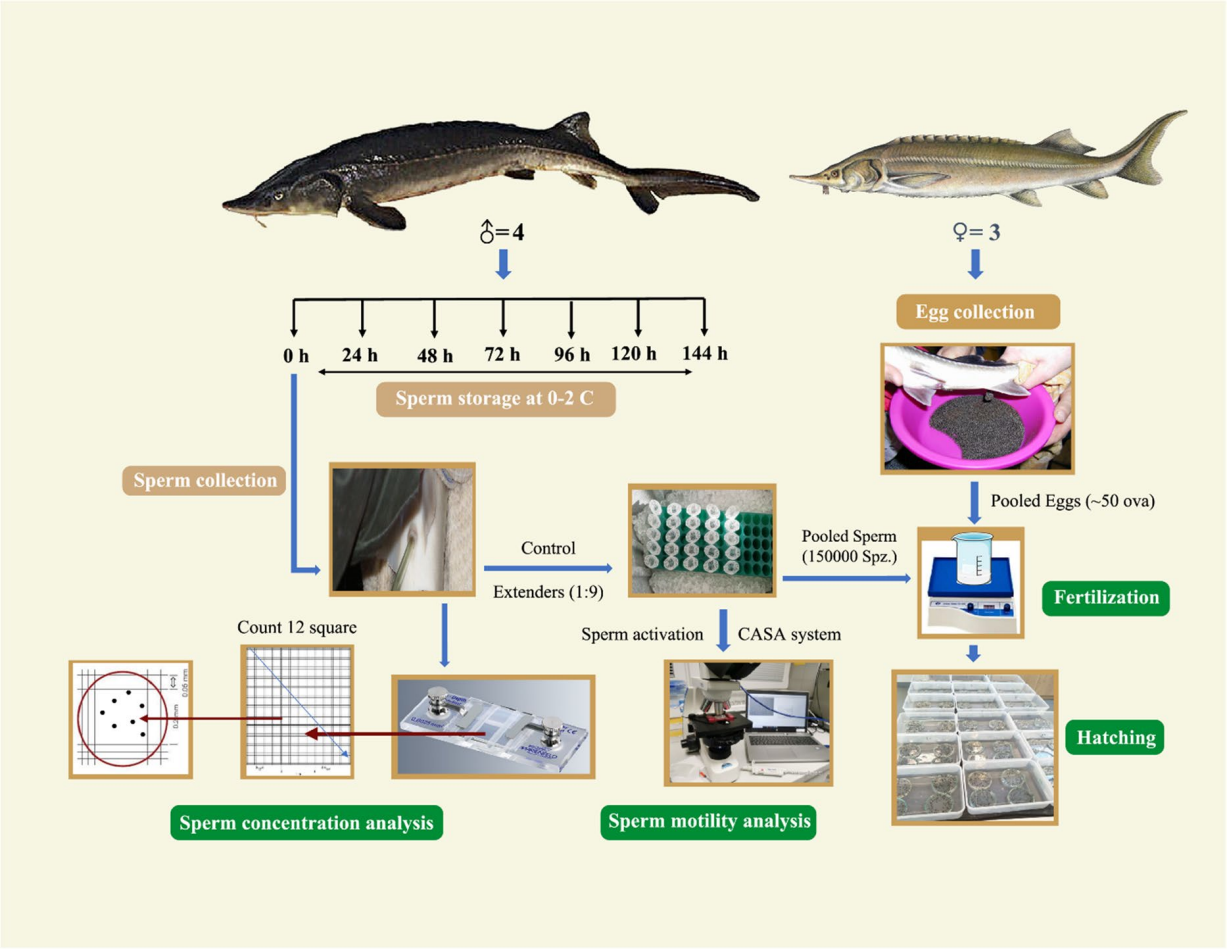
Schematic figures illustrate the experimental design of the study

In our previous experimental study, we designed 20 extenders based on analyzed seminal plasma parameters and past experimental knowledge of artificial seminal plasma formulations and finally optimized three extenders for our current study. The compositions of these three extenders were shown in Table S1. In the current study, collected sperm samples from individual males were taken into 15 mL plastic containers and then diluted with these three extenders, respectively by maintaining sperm with an extender ratio of 1:9. The final volume of sperm for each male was 5 mL (0.5 mL sperm + 4.5 mL extender), which was stored under an aerobic condition at 0–2 °C in a 15 mL plastic container. In our study, the extenders were only focused on the ionic composition but did not consider any antibiotics or sugar, or antioxidants, those might be considerable for our future study. The experimental design is depicted in Fig. 1, along with the time intervals during which sperm motility and velocity were assessed during sperm storage. Finally, the fertilization and hatching success of fresh and stored sperm were determined.

### Sperm concentration and seminal plasma analysis

The sperm concentration was evaluated by diluting individual sperm samples with physiological saline (0.9% NaCl *w*/*v*) at a ratio of 1:2000, and 10 µL of the suspension was placed on a Burker cell hemocytometer (Marienfeld, Germany) and left for 2–3 min for sperm to settle. The number of sperm was counted using an optical phase contrast condenser and an ISAS digital camera (PROISER, Spain) under an Olympus microscope BX 41 (4009) for the following fertilization assay. Each sperm sample, around 2 mL, was centrifuged for 15 min at 4 °C and 13,000 × g (Thermo Scientific, Fresco 21). Ion Selective Electrodes (ISE, Bayer HealthCare, USA) were utilized in potentiometry to measure the amounts of sodium (Na +), potassium (K +), and chloride (Cl^−^) ions. The concentration of calcium (Ca2 +) ions was measured using o-cresolphtwerein complexone absorption photometry (Moorehead and Biggs 1974). The Infinite M200 photometer (Tecan, Mânnedorf, Switzerland) was used to measure the protein concentration of seminal plasma using the bicinchoninic acid test (Thermo Fisher Scientific, Waltham, USA). Using a Freezing Point OSMOMAT 3000 (Gonotec, Germany), the osmolality of the seminal plasma was determined and reported as mOsmol/kg. The pH of seminal plasma was determined using a laboratory pH meter 320 (WTW, Germany).

### Assessment of sperm motility parameters

Sperm motility was measured as soon as possible after collection; samples with > 80% motility were used (*n* = 4) for analysis, whereas lower motility sperm samples were not considered for further analysis. Afterward, selected good motility sperm samples were diluted with appropriate extender (E1–E3) and checked sperm motility parameters at different time points (0, 1, 2, 3, 4, 5, and 6 days) during storage. Along with diluted samples, we measured the same motility parameters for undiluted sperm which was considered a control for comparing the motility kinetics of diluted sperm. To reduce spermatozoon adhesion to microscope slides, 0.25% Pluronic F-127 was added to a 10 µL activation mixture (2 mM CaCl_2_, 10 mM Tris–HCl, pH 8.0) (Shazada et al. 2024b) used for the activation of sperm motility, which was taken by a needle and mixed gently. Sperm motility was recorded using an ISAS digital camera (PROISER, Spain) fitted with uEye Cockpit software and set to 25 frames per s. The Olympus microscope BX 41 with a negative phase-contrast condenser and a 10 × objective lens was used to monitor the motility of the sperm. After recording, sperm motility was measured for 15 s post-activation at each storage time point and for each sperm sample, but the total recording was 30 s (750 frames). The Integrated System for Semen Analysis program (PROISER, Spain) was used to analyze the percentage of motile sperm as well as the spermatozoon curvilinear velocity over the actual path (VCL, µm/s) and straight-line velocity (VSL, µm/s). All samples were analyzed three times, and sperm that were slower than 10 µm/s were classified as non-motile. Every observation was made at room temperature.

### Fertilization, hatching, and malformation count

In a controlled, air-conditioned laboratory at 17 °C, the fertilization experiment involved developing and hatching larvae from the sterlet gametes in Petri dishes under a static tabletop setup. To minimize the variation in fertilization rates associated with egg quality, equal numbers of eggs from three females were collected and mixed. The diluted stored sperm with selected extenders from four males were pooled and used in the fertilization assay. Undiluted fresh and old sperm from similar males were also pooled and used as controls for fertilization and hatching analysis. A 25 mL beaker on a shaking table containing pooled eggs from three females (0.6 g, *c.* 50 ova), which were fertilized with pooled sperm (150,000 sperm; 3000 sperm/egg). Sperm was transferred to the bottom of the beaker near the ova using a pipette. Four beakers for each treatment were set on a shaking table, and 5 mL of dechlorinated water was given to each beaker and shaken for 2 min (150 rpm) at 17 °C. With extreme caution, the fertilized eggs were transferred from the four beakers into four Petri dishes (95 mL; 9 cm in diameter and 1.5 cm in depth). Every Petri dish was filled with dechlorinated water. Each Petri dish was set inside a small plastic box (13.5 cm × 10 cm × 6.5 cm), filled to one-third of its total capacity with 300 mL of dechlorinated water. One big plastic box measuring 28 cm by 21.5 cm by 7.5 cm was used to hold the four smaller boxes. The dechlorinated water was gradually changed 1 day after fertilization, and the water in the small plastic boxes was replaced daily until the embryos hatched. Non-developing embryos, or white eggs, were removed, and the water was exchanged daily after neurulation (3 days after fertilization). The fertilization rate was determined by dividing the number of viable eggs at the neurula stage by the total number of eggs recorded in the photographic records and the incubation period lasted for 6 days after activation or until hatching. The hatchlings were carefully counted and compared with the initial number of eggs in each Petri dish.

### Statistical analysis

The current study analyzed all data using Statistica v. 13 (TIBCO Software Inc., USA). Mean percentages of sperm motility, velocity, fertilization rate, hatching rate, and malformations are presented as mean ± SD. Residuals were tested for normality (Shapiro–Wilk test) and homogeneity of variance (plot of residuals *v.* predicted values). All the percentage data (sperm motility, fertilization, hatching, and malformation) were converted to arcsin root-square before analysis. A two-way factorial analysis of variance (ANOVA) model was performed to determine the effects of different extenders and sperm storage periods and their interaction on sperm motility, VCL, and VSL. When significant effects were detected, the model was decomposed into a lower-order one-way ANOVA model for each sperm storage period. A posteriori analysis (Tukey’s test) was performed for each sperm storage period and extender to determine the differences in sperm motility, VCL, and VSL. Alpha was set at 0.05 for significant differences. To compare fertilization, hatching, and malformation between freshly stripped and 6-day stored sperm, a *t* test was performed, and a comparison of different extenders was performed with one-way ANOVA with a posterior analysis (Tukey’s test).

## Results

### Sperm concentration, motility, and velocity

The sperm concentrations of the four selected males were 0.88 – 1.46 × 10^9^ spz mL^−1^. For the fertilization assay, pooled undiluted sperm was used as the fresh control, where the sperm concentration was 1.21 × 10^9^ spz mL^−1^ and the concentration of diluted pooled sperm with extenders (E1–E3) was recorded as 0.88 × 10^9^ spz mL^−1^, 1.42 × 10^9^ spz mL^−^1, and 1.46 × 10^9^ spz mL^−1^, respectively. Thus, the sperm volume used in the fertilization assay was determined according to their respective concentration value, e.g., undiluted fresh sperm (0.83 µL), diluted stored sperm with extender E1 (13.33 µL), E2 (12.66 µL), and E3 (14.92 µL) to fertilize 0.6 g (~ 50) eggs.

**Fig. 2:**
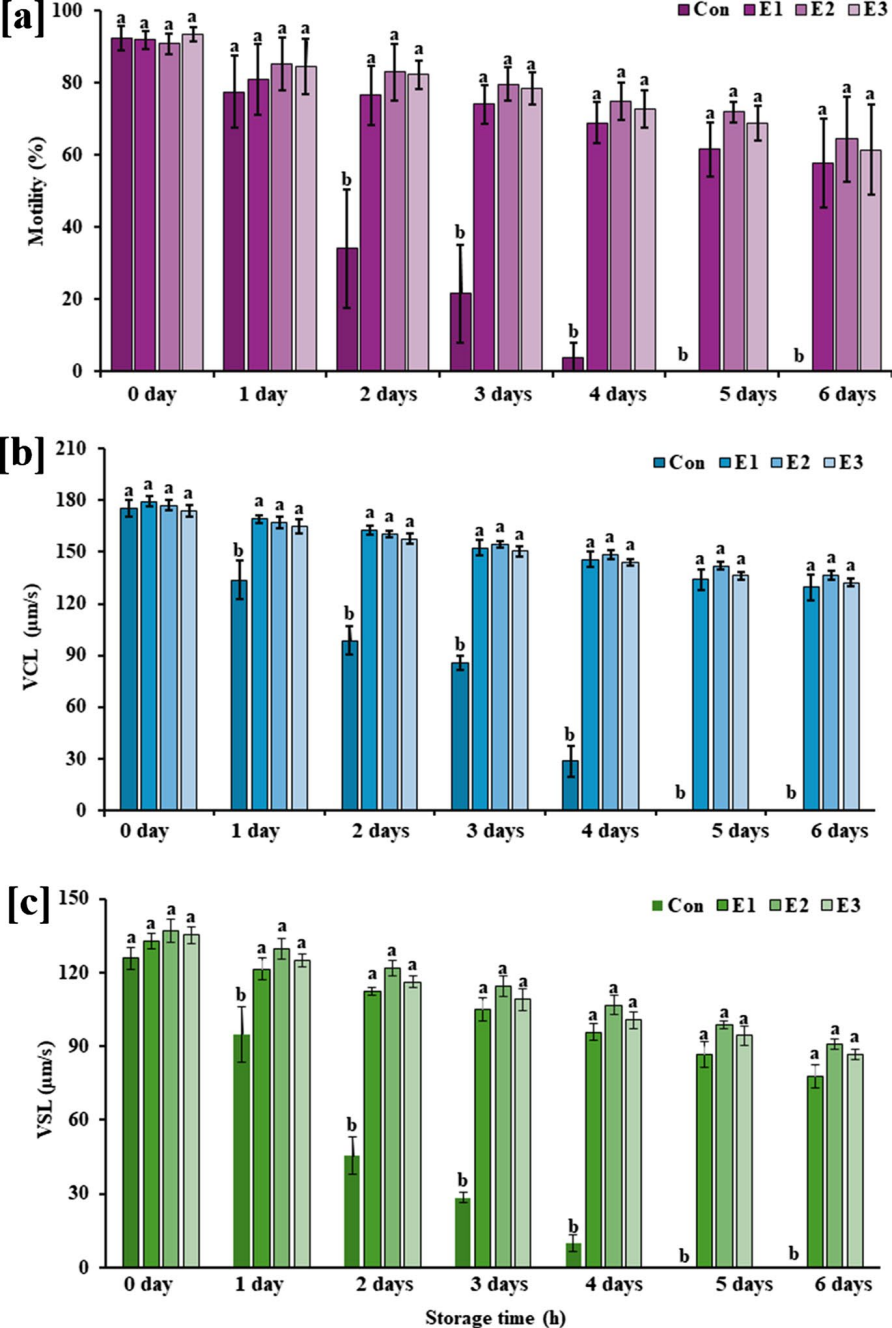
One-way ANOVA showing the effects of extenders (E1–E3) on **a** spermatozoa motility, **b** curvilinear velocity (VCL), and **c** straight-line velocity (VSL) at different sperm storage periods (1–144 h) (*n* = 4). Data are presented as mean ± SD. Treatments without a common superscript differ significantly (*P* < 0.001)

The two-way ANOVA showed significant effects of extenders (E1–E3), storage time (0 – 6 days) and extender × storage time interaction on sperm motility (*F* = 400.3, *P* < 0.001; *F* = 115.6, *P* < 0.001; *F* = 28.6, *P* < 0.001), VCL (*F* = 529.9, *P* < 0.001; *F* = 162.3, *P* < 0.001; *F* = 33.4, *P* < 0.001) and VSL (*F* = 24.98, *P* < 0.0001; *F* = 10.34, *P* < 0.0001; *F* = 1.78, *P* < 0.001). Then, the model was decomposed into a series of lower-order ANOVA models (one-way ANOVA) to study the main effects, including storage time and the effects of the extenders on sperm motility performances. The mean percentages of fresh sperm motility in control (undiluted sperm) and diluted sperm with extenders ranged from 84.35 to 96.65%. Therefore, the present study did not determine male effects on sperm motility and velocity. However, the percentage of sperm motility gradually decreased in three extenders with increasing storage periods. In the control group, motility was achieved in 3.77% of sperm at 4 days post-storage. After that, it became immotile at 5 days post-storage (Fig. 2a). Among the extenders examined, E2 was the most suitable storage medium, in which sperm motility was 64.34% at 6 days post-storage (Fig. 2a; Table S2). For VCL and VSL, as for sperm motility, a similar trend was observed, where the lowest VCL and VSL of sperm were 28.54 µm/s and 9.88 µm/s for the control group at 4 days post-storage (Fig. 2b, c; Table S3 & S4). The highest VCL and VSL were measured at 136.49 µm/s and 90.91 µm/s in diluted sperm with E2 at 6 days post-storage (Fig. 2b, c; Table S3 & S4).

### Effects of extenders on sperm motility performances

Our proposed extenders had significant effects on sperm motility (*P* < 0.001), VCL (*P* < 0.001), and VSL (*P* < 0.0001). Extenders significantly affected sperm motility at 2 days (*F* = 43.07, *P* < 0.0001), 3 days (*F* = 74.55, *P* < 0.001), 4 days (*F* = 114.70, *P* < 0.001), 5 days (*F* = 151.40, *P* < 0.001), and 6 days (*F* = 70.48, *P* < 0.0001) post-storage (Fig. 2a). The VCL of undiluted sperm was significantly lower than the diluted sperm groups (E1–E3) at 1 day (*F* = 7.06, *P* < 0.001), 2 days (*F* = 45.11, *P* < 0.0001), 3 days (*F* = 89.77, *P* < 0.001), 4 days (*F* = 120.90, *P* < 0.001), 5 days (*F* = 393.50, *P* < 0.001), and 6 days (*F* = 271.8, *P* < 0.001) post storage (Fig. 2b). The VSL of undiluted and diluted sperm was also significantly varied at 1 day (*F* = 5.760, *P* < 0.001), 2 days (*F* = 70.36, *P* < 0.0001), 3 days (*F* = 102.10, *P* < 0.001), 4 days (*F* = 168.40, *P* < 0.001), 5 days (*F* = 196.60, *P* < 0.001), and 6 days (*F* = 248.20, *P* < 0.001) post storage (Fig. 2c). There were no significant variations in VCL and VSL among E1–E3 treatment groups at 0 to 6 days post-storage (*P* > 0.05).

### Effects of storage time on sperm motility performances

Storage time had significant effects on undiluted and diluted (E1–E3) sperm motility performances (*P* < 0.001), VCL (*P* < 0.001), and VSL (*P* < 0.0001). In undiluted sperm, sperm motility significantly varied among different storage times (*F* = 222.00, *P* < 0.001), where sperm motility sharply declined from 92.33 to 3.77% at 4 days post storage (Table S2). After that, it became immotile (Fig. S1a). Sperm motility also varied significantly at different storage times in diluted sperm group E1 (*F* = 15.36, *P* < 0.0001), E2 (*F* = 4.55, *P* < 0.001), and E3 (*F* = 6.517, *P* < 0.0001). Sperm motility gradually declined from 91.83 to 57.56% for E1, 90.80 to 64.34% for E2, and 93.50 to 61.40% for E3 at 6 days post storage (Fig. S1a; Table S2). As in sperm motility, different storage times had significant effects on VCL (*F* = 102.60, *F* = 15.73, *F* = 29.62, *F* = 26.63; *P* < 0.001) and VSL (*F* = 78.66, *F* = 23.21, *F* = 21.45, *F* = 27.46; *P* < 0.001) in undiluted and diluted sperm groups (E1–E3) (Fig. S1b and S1c).

### Fertilization, hatching, and malformation

**Fig. 3:**
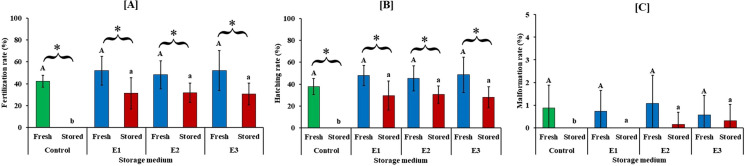
Fertilization (**a**), hatching (**b**), and malformation (**c**) rate of freshly stripped and non-diluted and diluted 144 h stored sperm. Data are presented as mean ± SD. The asterisk (*) sign on the bars indicates significant differences between fresh sperm and sperm stored for a short period (*t* test). The same letters on the bars indicate no significant differences among different extenders for freshly stripped pooled sperm (uppercase letters) and 144 h stored diluted and non-diluted pooled sperm (lowercase letter) (one-way ANOVA)

The fertilization and hatching rate of freshly stripped pooled sperm and 6 days stored pooled sperm significantly varied for all treatment groups (*t* test, *P* < 0.05) (Fig. 3a, b). Pooled undiluted sperm (control) stored for 6 days had zero fertilization and hatching rate. Fertilization and hatching of pooled diluted sperm with different extenders (E1–E3) ranged from 30.67 to 31.66% and 27.95 to 30.50%, respectively, which were significantly different compared to the pooled non-diluted sperm (control) (one-way ANOVA, *P* < 0.01). There was no significant difference in malformation of larvae between freshly stripped pooled sperm and 6 days stored pooled sperm (*t* test, *P* > 0.05). Pooled undiluted (control) and diluted sperm with E1 stored for 6 days had zero malformation rates, which was significantly different compared to E2 and E3 (one-way ANOVA, *P* < 0.05) (Fig. 3c).

## Discussion

In the current study, we examined sterlet sperm diluted with three selected extenders (16, 20, and 24 mM NaCl, 1 mM KCl, 0.1 mM CaCl2, 10 mM Tris, pH 8.0 with osmolarity of 46, 55, and 62 mOsm/kg). These showed better sperm motility performance than undiluted sperm. It was also noted that these three extenders showed significantly higher motility, VCL, and VSL at 6 days post-storage than the control group, which became immotile after 4 days post-storage. Thus, we considered these three extenders containing higher osmolarity and lower K^+^ as the most suitable storage medium for the short-term preservation of sterlet sperm. As demonstrated by various studies (Gallis et al. 1991; Cosson and Linhart 1996; Toth et al. 1997; Linhart et al. 2003), the inhibition of sperm motility can be triggered by K^+^ ions, whereas sperm are immotile in seminal plasma. The inhibitory concentration of K^+^ ions was also estimated at 0.75 mM in shortnose sturgeon (*Acipenser brevirostrum*) (Wayman 2003), 0.5 mM in American paddlefish (*Polyodon spathula*) (Linhart et al. 2002) and 0.35 mM in sterlet (Alavi et al. 2011). However, the inhibitory effects of K^+^ are overcome by Ca^2+^ ions, which are prominent in sperm activation in various fish species (Linhart et al. 2002; Cosson 2004; Alavi et al. 2011). A decrease in sperm motility performance was observed upon increasing the K^+^ ions in the extender (≥ 3 mM) in sterlet (Shazada et al. 2024b). As sperm motility initiation in sturgeons is regulated by Ca^2+^–K^+^ antagonistic activity (Cosson and Linhart 1996; Alavi et al. 2011), it is plausible that the activation medium needs Ca^2+^ ions to enhance sperm motility performance.

According to previous studies it has been noted that the osmolarity of sterlet seminal plasma is lower than other bony fishes due to spontaneous urine contamination (Dzyuba et al. 2014; Alavi et al. 2019; Shazada et al. 2024b). The osmolarity and seminal plasma composition of the sperm samples of good quality sperm in a previous study were reported as Na^+^ = 17.56 ± 10.12, K^+^ = 2.48 ± 1.14, Ca^2+^ = 0.11 ± 0.06, and Cl^−^ = 6.20 ± 3.42 mmol/L with an osmolarity of 35.60 ± 20.70 mOsm/kg (Shazada et al. 2024b). The present study agrees with a previous survey of sterlet, where similar ionic compositions and osmolarity were observed in good seminal plasma (Xin et al. 2020). The osmolality of bad-quality sperm is usually 14.66 ± 1.20 mOsmol/kg, while the osmolality of good-quality sperm is 33.66 ± 1.33 mOsmol/kg (Xin et al. 2020). However, the ionic composition and osmolarity of good-quality sperm significantly differ from low-quality sperm, which may not be appropriate for short-term storage.

The present study revealed that the percentage of sperm motility declines with an increasing storage period. Generally, during the short-term storage, undiluted sperm retains high values of sperm motility and velocity over a day and then declines with increasing storage period. However, sperm diluted with appropriate extenders can retain up to 57.56 to 64.34% sperm motility at 6 days post storage. It has been shown that immature sperm maintained in urine or seminal plasma from the Wolffian duct develops the ability for motility in sterlet testicular sperm (Dzyuba et al. 2014). Additionally, the motility of poor-quality sperm can increase when incubated in the seminal plasma of good-quality sperm (Xin et al. 2020). Sperm motility is maintained throughout the storage period when they are incubated in an isotonic state relative to seminal plasma (Xin et al. 2020). This is because sperm can regenerate ATP in an isotonic environment, which is necessary for sperm to overcome the axoneme, the sperm’s motility mechanism (Cejko et al. 2019; Linhart et al. 2008; Billard et al. 1995). Sperm motility features that affect the fertility of sperm cells include sperm velocities (VCL and VSL), which are vital components for breeding success (Linhart et al. 2008; Tuset et al. 2008; Viveiros et al. 2010; Gallego et al. 2017). The maximum VCL (136.49 µm/s) and VSL (90.91 µm/s) were obtained in this study for diluted sperm with E2 at 6 days post-storage, while the lowest VCL (28.54 µm/s) and VSL (9.88 µm/s) were found with undiluted sperm at 4 days post-storage. In the present study, sperm velocity dropped at 4 days post-storage without extenders, indicating that a suitable extender is required to maintain sperm velocity for short-term sperm storage.

The current investigation also found that storage duration significantly impacted the motility kinetics of all sperm, regardless of whether specific extenders were used. These findings align with earlier research on motility kinetics (Shaliutina et al. 2013; Sarosiek et al. 2014; Cheng et al. 2021). However, the storage period significantly affected undiluted and diluted sperm motility performances. In undiluted sperm, the percentage of motility sharply declined from 92.33 to 3.77% at 4 days post-storage, and after that, it became immotile. In contrast, diluted sperm with E1–E3 showed a gradual decrease in sperm motility performances during each storage (0 to 6 days post-storage). Like sperm motility, different storage times significantly affected VCL and VSL in undiluted and diluted sperm groups (E1–E3). It was noted that damage to sperm morphology and reduced energy storage are associated with storage effects on sperm motility kinetics (Contreras et al. 2020; Bobe and Labbe 2010; Billard et al. 1995).

In the present study, we observed that the fertilization and hatching rate using freshly ovulated sperm (fresh control) and 6 days stored pooled diluted sperm showed a significant effect for all treatment groups. When compared to undiluted pooled fresh sperm, the rates of fertilization and hatching using pooled diluted sperm with various extenders (E1–E3) varied from 30.67 to 31.66% and 27.95 to 30.50%, while undiluted stored sperm had zero fertilization and hatching rate. The fertilization and hatching rates of freshly ovulated eggs with diluted sperm were 48.09 to 52.01% and 45.25 to 48.54%. We observed that about 50% of fertilization and hatching success were achieved for all treatment groups in this study, which might be due to the quality of sterlet eggs, the number of sperm used for fertilization, or the dilution of sperm with water. To enable homogenous fertilization, a pool of sperm was typically used to lessen issues with sperm-egg interaction (Linhart et al. 2005). Previous studies indicated that the impact of female differences on fertilization and hatching outcomes could have been mitigated by pooling eggs from several females (Dzyuba et al. 2012; Xin et al. 2018). According to Iegorova et al. (2018), the quantity of sperm (1,500,000 sperm) in the 3 mL of water during activation was ideal for preventing polyspermy and achieving a highly satisfactory fertilization and hatching rate. According to a previous study, a technique to minimize the likelihood of polyspermy during egg fertilization is to dilute sperm at ratios of 1:100 to 1:200 with water (Dettlaff et al. 1993). Linhart et al. (2020) found that neurulation and hatching in sterlet gradually decreased with a decrease of the sperm and egg ratio from 6250 to 114; therefore, the 3000 sperm per egg used in the present study was sufficient to achieve optimum fertilization and hatching success. Linhart et al. (2020) also found that the ratio of 1:4 between eggs and water was insufficient to provide adequate fertilization; therefore, we used higher values ​​in our study, which was 1:8. In the present study, instead of placing sperm directly on the eggs during the fertilization assay, the sperm was inserted by pipette into the bottom of the beaker near the eggs to activate the gametes. As a result, from the start of the fertilization process, the sperm in the water was homogeneous. To achieve more homogeneity and lessen the adherence of the eggs to one another, it was also crucial to gently mix the eggs with sperm and activation medium. At the same time, beakers with samples were placed on a shaker table and set to 150 rpm for homogeneous mixing of eggs with sperm and activation solution (Cheng et al. 2020).

In the present study, the malformation rate was observed less than 1.07% for all treatments, including fresh sperm (diluted and undiluted) and diluted sperm with extenders after 6 days post-storge, but interestingly the fresh sperm showed little bit higher malformation rate than short-term stored sperm, which are negligible. In this case, it might be possible that during storage only healthy individuals of sperm can sustain after 6 days post-storage, but fresh sperm contain all together. Generally, sperm aging during short-term storage typically results in reduced sperm quality, including DNA fragmentation, mitochondrial membrane potential, and plasma membrane integrity (Shaliutina et al. 2013; Trigo et al. 2015). This could lead to a rise in the number and rate of deformed larvae following fertilization (Gosálvez et al. 2014; Herráez et al. 2017).

## Conclusion

Short-term sperm storage is crucial in hatchery conditions because it can maintain sperm in quiescence, essential for artificial egg fertilization. The present study demonstrated that sterlet sperm diluted with our proposed extenders can successfully maintain sperm functionality up to 6 days post-storage and achieve 30.67–31.66% fertilization and 27.95–30.50% hatching success when fertilized with freshly stripped eggs after 6 days. The results of fertilization and hatching rate of our study did not exceed 40%; therefore, further studies should be performed considering its composition, including sugars, antioxidants, or antibiotics. Utilizing high-quality sperm minimizes the possibility of experiencing a low rate of fertilization. Maintaining sperm quality by using suitable extenders during short-term preservation could guarantee better fertilization outcomes and preserve more genetic variation, particularly for endangered species whose gametes and broodstock are typically limited throughout each striping cycle.

## Supplementary Information

Below is the link to the electronic supplementary material.

Supplementary file1 (DOCX 66 KB)

## Data Availability

No datasets were generated or analysed during the current study.
