# Supplementary material for: Effect of appropriate extenders to maintain sperm functionality during short-term storage of sterlet (Acipenser ruthenus) sperm with fertilization assay under hatchery conditions
Source: Fish Physiol Biochem. 2024 Dec 2;51(1):14. doi: 10.1007/s10695-024-01413-7 (PMC11611934; doi:10.1007/s10695-024-01413-7)
Supplement: Supplementary file 1 — Supplementary file1 (DOCX 66 KB) [file 10695_2024_1413_MOESM1_ESM.docx]

**Supplementary Information**

**Effect of appropriate extenders to maintain sperm functionality during short-term storage of sterlet (*Acipenser ruthenus*) sperm with fertilization assay under hatchery conditions**

Nururshopa Eskander Shazada^1,2^, Mohammad Abdul Momin Siddique^1,3^, Songpei Zhang^1^, Zhijun Ma^1^, Marek Rodina^1^, Otomar Linhart^1*^

^1^ University of South Bohemia in **České Budějovice,** Faculty of Fisheries and Protection of Waters, South Bohemian Research Center of Aquaculture and Biodiversity of Hydrocenoses, Vodňany 38925, Czech Republic

^2^ Department of Biotechnology and Genetic Engineering, Noakhali Science and Technology University, Noakhali 3814, Bangladesh

^3^ Department of Oceanography, Noakhali Science and Technology University, Noakhali 3814, Bangladesh

**Running title:** Short-term storage of sterlet sperm under hatchery conditions

**^*^Correspondence:** Prof. Otomar Linhart, DrSc

University of South Bohemia in **České Budějovice,** Faculty of Fisheries and Protection of Waters, South Bohemian Research Center of Aquaculture and Biodiversity of Hydrocenoses, Research Institute of Fish Culture and Hydrobiology, **Zátiší** 728/II, Vodňany 38925, Czech Republic; E-mail: [linhart@frov.jcu.cz](mailto:linhart@frov.jcu.cz)

**Table S1.** Composition of extenders used for short-term storage of sterlet (*Acipenser ruthenus*) sperm in the present study.

| **Name of extender** | **Composition of ions (mM)** | **Osmolarity (mOsm/Kg)** | **pH** |
| --- | --- | --- | --- |
| Extender 1 (E1) | 16 mM NaCl + 1 mM KCl + 0.1 mM CaCl2 + 10 mM Tris HCl | 46 | 8 |
| Extender 2 (E2) | 20 mM NaCl + 1 mM KCl + 0.1 mM CaCl2 + 10 mM Tris HCl | 55 | 8 |
| Extender 3 (E3) | 24 mM NaCl + 1 mM KCl + 0.1 mM CaCl2 + 10 mM Tris HCl | 62 | 8 |

**Table S2.** Mean percentage of sperm motility of sterlet sperm with different storage periods.

| Storage time (days) | Undiluted sperm (Con) | Diluted sperm with extender 1 (E1) | Diluted sperm with extender 2 (E2) | Diluted sperm with extender 3 (E3) |
| --- | --- | --- | --- | --- |
| 0 | 92.33 | 91.83 | 90.80 | 93.50 |
| 1 | 77.45 | 80.95 | 85.24 | 84.53 |
| 2 | 34.04 | 76.57 | 82.91 | 82.20 |
| 3 | 21.50 | 74.01 | 79.58 | 78.49 |
| 4 | 3.77 | 68.86 | 74.97 | 72.69 |
| 5 | 0.00 | 61.50 | 71.86 | 68.92 |
| 6 | 0.00 | 57.57 | 64.34 | 61.40 |

**Table S3.** Curvilinear velocity (VCL µm/s) of sterlet sperm with different storage periods.

| Storage time (days) | Undiluted sperm (Con) | Diluted sperm with extender 1 (E1) | Diluted sperm with extender 2 (E2) | Diluted sperm with extender 3 (E3) |
| --- | --- | --- | --- | --- |
| 0 | 175.45 | 179.54 | 177.15 | 174.16 |
| 1 | 133.83 | 169.18 | 166.99 | 164.80 |
| 2 | 98.73 | 162.81 | 160.63 | 157.75 |
| 3 | 85.50 | 152.46 | 154.67 | 150.38 |
| 4 | 28.54 | 145.86 | 148.63 | 144.13 |
| 5 | 0.00 | 134.03 | 141.94 | 136.15 |
| 6 | 0.00 | 129.43 | 136.49 | 132.11 |

**Table S4.** Straight line velocity (VSL µm/s) of sterlet sperm with different storage periods.

| Storage time (days) | Undiluted sperm (Con) | Diluted sperm with extender 1 (E1) | Diluted sperm with extender 2 (E2) | Diluted sperm with extender 3 (E3) |
| --- | --- | --- | --- | --- |
| 0 | 125.89 | 133.03 | 137.13 | 135.27 |
| 1 | 94.86 | 121.44 | 129.78 | 124.95 |
| 2 | 45.39 | 112.42 | 121.83 | 116.22 |
| 3 | 28.44 | 105.15 | 114.38 | 109.14 |
| 4 | 9.88 | 95.77 | 106.82 | 100.68 |
| 5 | 0.00 | 86.58 | 98.66 | 94.25 |
| 6 | 0.00 | 77.72 | 90.91 | 86.51 |


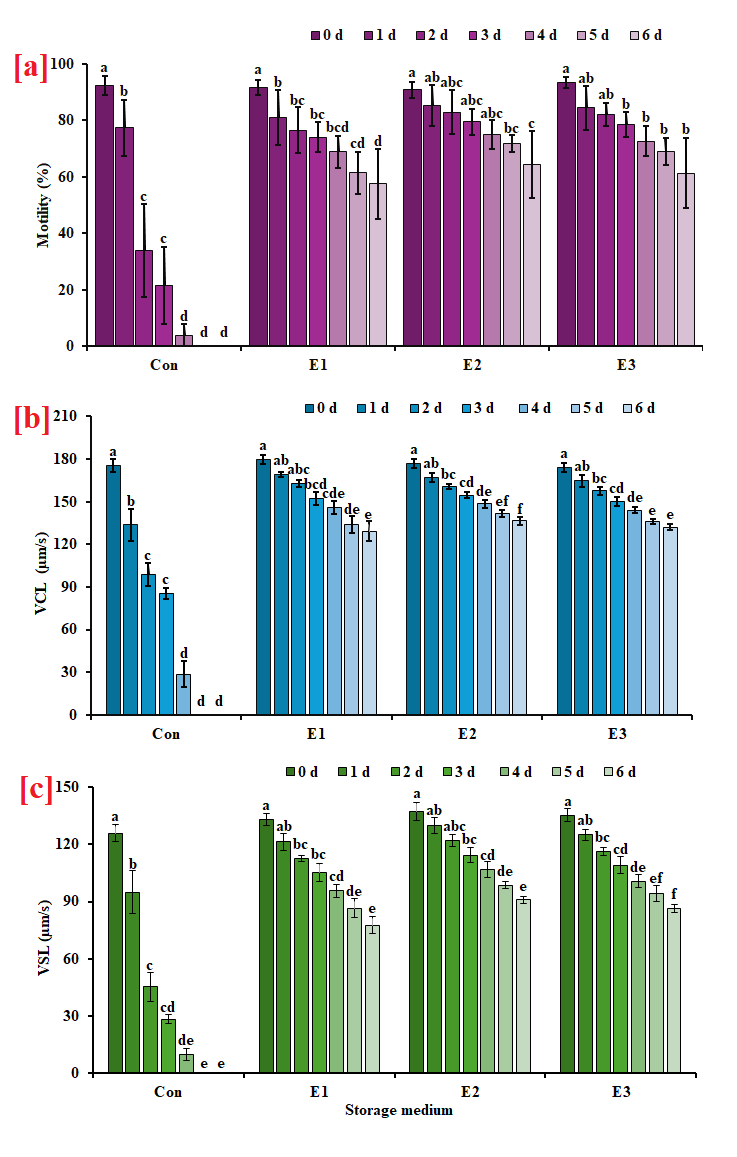


**Fig. S1** One-way ANOVA showing the effects of different sperm storage periods (1, 24, 48, 72, 96, 120, and 144 h) on (a) spermatozoa motility, (b) curvilinear velocity (VCL), and (c) straight-line velocity (VSL) with selected extenders (E1–E3) (*n*= 4). Data are presented as mean ± SD. Treatments without a common superscript differ significantly (*P*<0.001).
